# A concordance between patent and trademark classes to link technologies to markets

**DOI:** 10.1038/s41597-026-07422-w

**Published:** 2026-05-23

**Authors:** Milad Abbasiharofteh, Carolina Castaldi, Sergio Petralia

**Affiliations:** 1 Aalborg University Business School, Faculty of Social Sciences and Humanities, 9220 Aalborg, Denmark; 2 Utrecht University, Department of Human Geography and Spatial Planning Utrecht, 3584 Utrecht, CB The Netherlands

**Keywords:** Technology, Intellectual-property rights, Economics

## Abstract

Patent data is the preferred source of information for tracking technological change. However, the economic impact of patented technologies remains unclear unless patent data is linked to other data, which can reveal the mechanisms through which new technology diffuses. One such data source comes from the trademark filings that accompany the market introduction of new goods and services. Patent and trademark data can be combined to link given technologies to specific markets. Yet, differences in their classification systems represent a challenge. To enable linking patent and trademark data, we develop, validate and share a novel concordance between technology classes in patent records and market classes in trademark records. The concordance can be used to track the diffusion of patented technologies at the technology, firm, region, or country level, with many relevant applications in research and policy analyses of innovation.

## Background & Summary

The information contained in patent documents has been extensively used by scholars and policymakers to shed light on fundamental aspects of technological change and inform innovation policy^1–3^. At the same time, assessing the value of patented technologies for firms, regions and society at large remains challenging given the fact that patents only signal technological inventions, whose potential and economic impact is highly uncertain and strongly heterogeneous. Economic returns from new technologies often materialize through adoption, following complex processes of technology diffusion and translation of invention into actual new goods and services.

Understanding how patented technologies find their way into commercialized innovation is one way to study the impact of new technologies. Such investigations should rely on complementary data that unveil these downstream phases of the innovation process. Trademark documents are emerging as a data source with the potential to help in this direction^4,5^. New trademarks are filed by companies when introducing a product innovation into the market^6,7^. Many companies file patents and trademarks in a complementary way, to cover different outputs of their innovation processes^8^. As data, trademark documents have many similarities to patent documents. Both intellectual property rights are filed at offices that process them against formal requirements, and all filings are published in online repositories that include information about the filer, the date of filing, the address of the filer, the outcome of the filing procedure, a text description, and much more. Against this background, earlier research has developed methods that draw on technology classes to extract relevant information in patent documents^9–11^ and to identify topics in the textual content of trademark filings^12^. Moreover, scholars have examined the relationship between inventive activities, measured through patenting, and commercialization, measured through trademarks, to analyse evolving business models and competition strategies^13^.

However, the two data sources also have differences that form challenges for analyses that combine them. In particular, patent and trademark records rely on specific classification systems, namely technology and market classes. Linking the two types of classes is instrumental in combining the two data types, but a concordance is currently missing. Concordances already exist between patents’ technology classes and industry codes^14^ and between trademarks’ market classes and industry codes^15,16^. Many concordances are deterministic and assume a simple one to one binary relationship between the entities on both ends. Probabilistic approaches go beyond indicating the presence or absence of a relationship and instead estimate the probability or intensity of a relationship between two entities. For example, Schmoch *et al*.^16^ use a probabilistic approach to link International Patent Classification (IPC) technology codes with the European classification system for economic activities (NACE codes). They derive their concordance from the patenting activities of 3000 firms classified by NACE codes. Zolas *et al*.^15^ also adopt a probabilistic approach to construct a concordance between trademark market classes and industry codes. These two examples of probabilistic approaches are already useful and they have already been integrated to indirectly link patent classes to trademark classes through industry codes^8^. Yet, this is a rather indirect way of linking patent to trademark classes. Therefore, further methodological efforts are needed to build a direct concordance between patent and trademark classes. Following the insights from related concordance work, we opt for a probabilistic approach that allows linking multiple technology classes to multiple market classes, with different weights to each market. This is the key objective of the work that we report here. The two main outputs that we share are the concordance itself (PAT2TM) and a more granular classification of market classes (Nice subclasses) than the standard 45 Nice classes included in trademark records.

Before describing the methodological steps and introducing the concordance, we outline its reuse value for research and policy. We envision three levels of analysis at which the concordance could be used: the technology level, the firm level, and the geographical level. First, one can track the diffusion of technologies patented in a given technology class by tracking trademark filings in the market classes linked to that technology class. Such analyses could extend previous work tracking patented technologies into industries^17^ or into occupations^18^. Frietsch *et al*.^19^ already showed the value of integrating patent and trademark data to trace the diffusion of key technologies. Our concordance complements those efforts by providing content-based matching of technology and market activity. Second, researchers interested in firm-level analysis could link the technology classes covered by the patent portfolios of companies to the market classes covered by their trademark portfolios. This approach could allow identifying gaps in technology commercialization, indicating in which markets companies could further exploit their technologies or comparing technology management strategies between firms or sectors by contrasting them to content-based linkages. In practice, different firms could specialize in technologies and markets that relate to each other. Third, one could study regional specialization in technology classes and their relationship with specialization in market classes, to uncover complementarities or specific innovation specializations^20,21^. Such applications seem particularly appealing for policy makers interested in specific innovation directions, for instance those related to environmental sustainability^22,23^. A content-based concordance like the one proposed here allows one to compare actual synergies to possible ones, indicating where technological specializations could be exploited further in the same region or how market specializations could feedback to local learning in relevant technological fields. Importantly, technology-market linkages do not always happen within the boundaries of one firm but can also realize in ecosystems of companies where some companies specialize in technological development and other companies specialize in translating those new technologies into new goods and services. Hence, a content-based concordance is more flexible than a concordance based on patent-trademark pairs found in firm portfolios. Nevertheless, we will also validate our content-based linkages against firm-level linkages happening in those select group of firms that file both patents and trademarks.

## Methods

This section describes the methodology for developing and evaluating a concordance between the technological classes assigned to patents and the market classes assigned to trademarks. We start by describing in detail the characteristics of the two main data sources, patent and trademark data.

### Properties of the Patent Data

We obtained detailed information on patents filed worldwide from PATSTAT Global (version: 2020), issued by the European Patent Office (EPO). PATSTAT includes comprehensive information on more than 100 million patent documents filed in more than 80 patent offices worldwide (including the legal event data from more than 40 authorities) (available at: https://www.epo.org/en/searching-for-patents/business/patstat). Of interest here is the classification of patents into technological domains using the Cooperative Patent Classification (CPC). The CPC is a standardized classification system built around a hierarchy of classes at different levels of detail, including broad sections with 1-digit codes that further expand to more than 200,000 8-digit classes (for a description of CPC codes, see https://www.uspto.gov/web/patents/classification/cpc/html/cpc.html). For instance, the 4-digit technological class G06F corresponds to ‘Electric digital data processing’ technologies within the broad section ‘Physics’ (G) but can be further specified up to class ‘G06F 1/025’, corresponding to ‘Digital function generators for functions having a two-valued amplitude, e.g., Walsh functions’. Empirical studies using patent data typically exploit 4-digit technological classes, which provide a good compromise between specifying the technological field with precision and ensuring meaningful comparisons of patent numbers^24–29^. Hence, we focus on developing a concordance for 4-digit CPC technology classes, for a total of 662 classes.

We further restricted the set of patent records according to two considerations. First, we used the DOCDB family identifier from PATSTAT to avoid counting duplicate patents filed at multiple patent offices. DOCDB families are defined by the European Patent Office and represent strict families based on the priority filing, ensuring that each invention is counted only once. Second, we only included patents filed between 1996 and 2020, to align with the time frame of trademark data availability. The final patent database contained 21,575,766 patent documents.

### Properties of the Trademark Data

We obtained open-access trademark data from the European Union Intellectual Property Office (EUIPO, see https://euipo.europa.eu/eSearch/), which manages Europe-wide trademark filings. The EUIPO started accepting trademark filings only in 1996^30^, therefore, we considered all trademarks filed between 1996 and 2020, for a total of 1,645,484 filings, accessed in March 2021.

The trademark filing process requires applicants to specify one or more markets in which they seek protection for their trademark. Markets relate to goods or services and are categorized according to the Nice international classification of goods and services (see https://www.wipo.int/en/web/classification-nice). The Nice classification system only specifies 45 broad classes, with classes 1-34 covering goods and classes 35-45 covering services. As such, its level of detail is very different from that provided by the CPC classification system. For instance, the ‘PyTorch’ trademark (filing number: 018235196) filed by the Linux Foundation for a machine learning framework for computer vision and natural language processing covers Nice class 9, corresponding to electronics and digital products, but also two service classes, 41 and 42, corresponding to training and research services.

Trademark filings also include a goods and services description, which is a text in which applicants describe the goods or services covered by the trademark. EUIPO strongly incentivizes applicants to use predefined terms by offering a fast-track application process. EUIPO’s trademark examiners developed a harmonised database (HDB) based on commonly received descriptors. The HDB comprises 85,233 terms that describe goods and services linked to a specific Nice class and HDB code (also known as external reference). Each HDB code may include multiple terms. For example, the HDB terms *packaging boxes of card* and *packaging cartons of card* fall under the same HDB code (i.e., *0000001*), denoted as *MASTER* and *VARIANT* terms. In total, the HDB contains 62,944 unique HBD codes assigned to 85,233 terms. HDB terms and their official translations into all EU languages (except Irish) are publicly available and embedded in the EUIPO’s online trademark fast-track application form (see http://euipo.europa.eu/ec2/?lang=en). They are regularly updated to reflect actual developments. HDB terms are relatively short and specific (the average number of words per term is 12.18). For the PyTorch example, the trademark record also includes several descriptors, in particular *Downloadable artificial intelligence software to facilitate machine learning*, corresponding to the HDB term 0076406, specific to Nice class 9.

We propose here to use the HDB terms to build a more detailed list of trademark classes than the 45 Nice classes. By doing so, we are able to specify trademark classes at a level of detail that is comparable to patent classes. In turn, this allows us to build a concordance linking the 662 4-digit CPC technology classes to 616 Nice subclasses. Such a concordance is more usable than a concordance linking 662 CPC technology classes to 45 Nice classes, which would link many technology classes to the same market classes. Hence, a concordance linking patent classes to trademark subclasses provides a more meaningful basis for studying the relationship between technologies and markets. It should also be noted that by relying on the HDB terms instead of the actual description provided by trademark applications we avoid potential biases that could arise from self-description by the applicants. At the same time, the fact that HDB terms capture the most commonly used terms means that they are relevant for all trademark applications, not only those going through the fast application process.

### A concordance between patent and trademark classes (PAT2TM)

Considering the specific data properties discussed in the previous section, our goal of constructing a taxonomy involved two objectives. A first and preliminary objective was to develop a list of detailed Nice subclasses that further specify the 45 Nice classes. We did this by leveraging the information on HDB terms (resulting in the HBS2Nicesub database). The second and main objective was to develop a concordance between 4-digit CPC patent classes and Nice subclasses, resulting in the PAT2TM concordance.

**Fig. 1:**
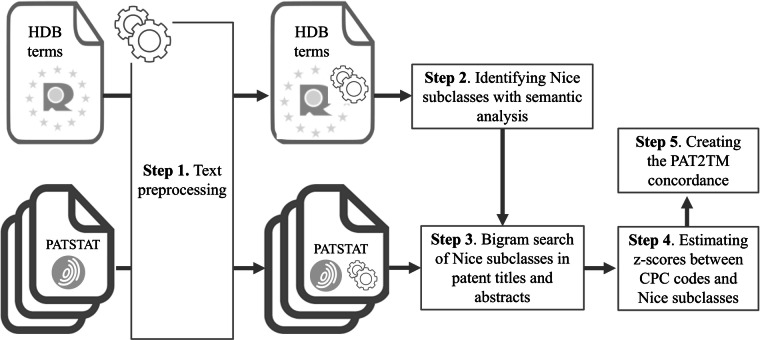
A schematic summary of our approach.

We took five main methodological steps for which Fig. 1 provides a schematic overview. In Step 1, we parsed the relevant text content of the patent records (title and abstract) and the list of HDB terms to prepare them for use in further steps. We followed what is customary in text-mining procedures. we: i) converted all words to lowercase, ii) omitted sentences that included negative words (e.g., not or non-), iii) removed English stop-words (e.g., the), iv) removed general words that did not carry a specific meaning (e.g., contain), v) removed numbers and punctuation, and vi) removed the suffix of each word and transform them to a corresponding base word (also known as stemming). Accordingly, the text ‘Machine learning software for analysis’ reads as ‘machin learn softwar analysi’.

In Step 2 we focused on defining Nice subclasses starting from the detailed information provided by HDB terms. To this end, we built on earlier efforts to create a fine-grained classification of EUIPO trademarks by linking applicant-provided descriptions to keywords using Levenshtein and Jaro Winkler matching^31^. However, our approach relied on a more advanced NLP technique (i.e., Doc2Vec) that does not depend solely on keyword similarity but also captures semantic similarity between terms. For example, the expressions *hosting solutions* and *virtual servers* receive low Levenshtein or Jaro-Winkler similarity scores even though they are semantically close to each other. To map a semantic space for each Nice class. We selected all parsed HDB terms listed under each Nice class in the trademark records. Table 1 shows the number of terms for each Nice class with the corresponding most frequent keywords (as stems).

**Table 1:** The number of HDB terms for each Nice class and the most frequent terms (stems).

Nice class	Type	Number of terms	Number of Nice subclasses	Top five keywords (stems)
1	Goods	3791	13	chemic, composit, manufactur, food, water
2	Goods	820	15	paint, natur, color, protect, metal
3	Goods	1309	19	cosmet, skin, lotion, cream, impregn
4	Goods	485	12	metal, briquett, oil, addit, concret
5	Goods	1967	18	medic, agent, pharmaceut, cream, care
6	Goods	1798	18	metal, rail, hook, plug, roof
7	Goods	3714	18	machin, cut, part, print, electr
8	Goods	852	14	hand, drill, saw, blade, bit
9	Goods	5761	14	electr, apparatus, applic, comput, download
10	Goods	2145	19	medic, apparatus, mask, protect, devic
11	Goods	2291	11	apparatus, electr, water, instal, sanitari
12	Goods	1621	16	vehicl, land, air, carriag, locomotiv
13	Goods	1336	8	firearm, explos, pyrotechn, gas, munition
14	Goods	1264	19	jewelri, watch, clock, timepiec, gem
15	Goods	456	3	musical, instrument, string, keyboard, drum
16	Goods	365	16	pap, book, print, offic, art
17	Goods	1326	15	gum, gutta, rubb, asbesto, mica
18	Goods	1956	10	leather, anim, skin, hide, imitat
19	Goods	705	17	furnitur, mirror, pictur, frame, display
20	Goods	968	12	utensil, container, household, kitchen, clean
21	Goods	278	16	small, hand, tool, cutleri, fork
22	Goods	664	12	cordag, string, net, tent, tarpaulin
23	Goods	509	14	yarn, thread, textil, cloth, manufactur
24	Goods	621	12	fabric, textil, cloth, manufactur
25	Goods	963	12	pant, short, sock, tror, wear
26	Goods	351	9	textil, cloth, hair, button, ornament
27	Goods	122	3	floor, carpet, cover, tile, wall
28	Goods	1460	12	set, toy, apparatus, game, play
29	Goods	1198	8	chees, pickl, potato, preserv, sausag
30	Goods	1722	19	food, flavour, confectioneri, flour, cake
31	Goods	867	16	fresh, fruit, edibl, anim, plant
32	Goods	188	10	drink, beverag, beer, water, alcohol
33	Goods	145	10	alcohol, beverag, liqueur, drink, whiski
34	Goods	183	14	pipe, tobacco, cigarett, electron, cigar
35	Services	1972	18	busi, advertis, manag, commerci, equip
36	Services	1682	12	financi, manag, administr, inform, brokerag
37	Services	2070	12	mainten, repair, build, instal, apparatus
38	Services	795	19	communic, data, transmiss, network, comput
39	Services	1214	15	transport, good, rental, passeng, travel
40	Services	946	17	fur, food, gas, mould, oil
41	Services	2510	13	educ, cours, organ, event, show
42	Services	2170	15	design, comput, develop, softwar, program
43	Services	352	10	hire, rental, bar, cater, drink
44	Services	877	19	inform, medic, field, advisori, breed
45	Services	464	12	inform, advisori, date, social, support

Within each Nice class we aimed to cluster HDB terms by identifying semantic associations between them. We did so by applying an extension of the Word2Vec algorithm^32^ called Doc2Vec^33^, which allows measuring semantic distance between HDB terms in a high-dimensional vector space. An essential advantage of HDB terms is that they are Nice class specific. Hence, we could apply Doc2Vec to text specific to each Nice class, which prevented erroneous associations between terms across disparate contexts and allowed similar associations in different market classes. Consequently, our approach could uncover the semantic association of the keywords “learn”, “machin” and “softwar” in Nice class 9 (electronics and digital applications) as different from the same association in Nice class 41 (education and training services).

We used the K-means clustering algorithm to group HDB terms based on their associations within each Nice class-specific semantic space. To specify the number of clusters for each Nice class, we used the information on the sum of squared distances, known as the elbow method. In other words, we plotted the explained variation and the number of clusters, and selected the point where the curve shows a significant bend to determine the optimal number of clusters. Each cluster of HDB terms identified a subclass within the given Nice class, for 616 Nice subclasses. We labeled each Nice subclass with a four-digit code listing the Nice class, the subclass number out of the total number of subclasses, and the relative size of the Nice subclass. For example,we identified 14 subclasses in Nice class 9 (electronics and digital applications). Nice subclass ‘9_5/14_0.12’ is characterized by the keywords (stems) *softwar, comput, network*, is the 5th subclass out of a total of 14 subclasses, and includes 12% of the HDB terms associated with Nice class 9.

In Step 3, we matched the CPC technology classes with the newly created Nice subclasses. The approach involved searching for the HDB terms of a Nice subclass in patent titles and abstracts. N-grams search is a widely used approach in empirical patent studies^3,25,26^. We chose to use bigrams because they provide more contextual information than unigrams, while ensuring a relatively higher number of matches than trigrams^18^.

**Fig. 2:**
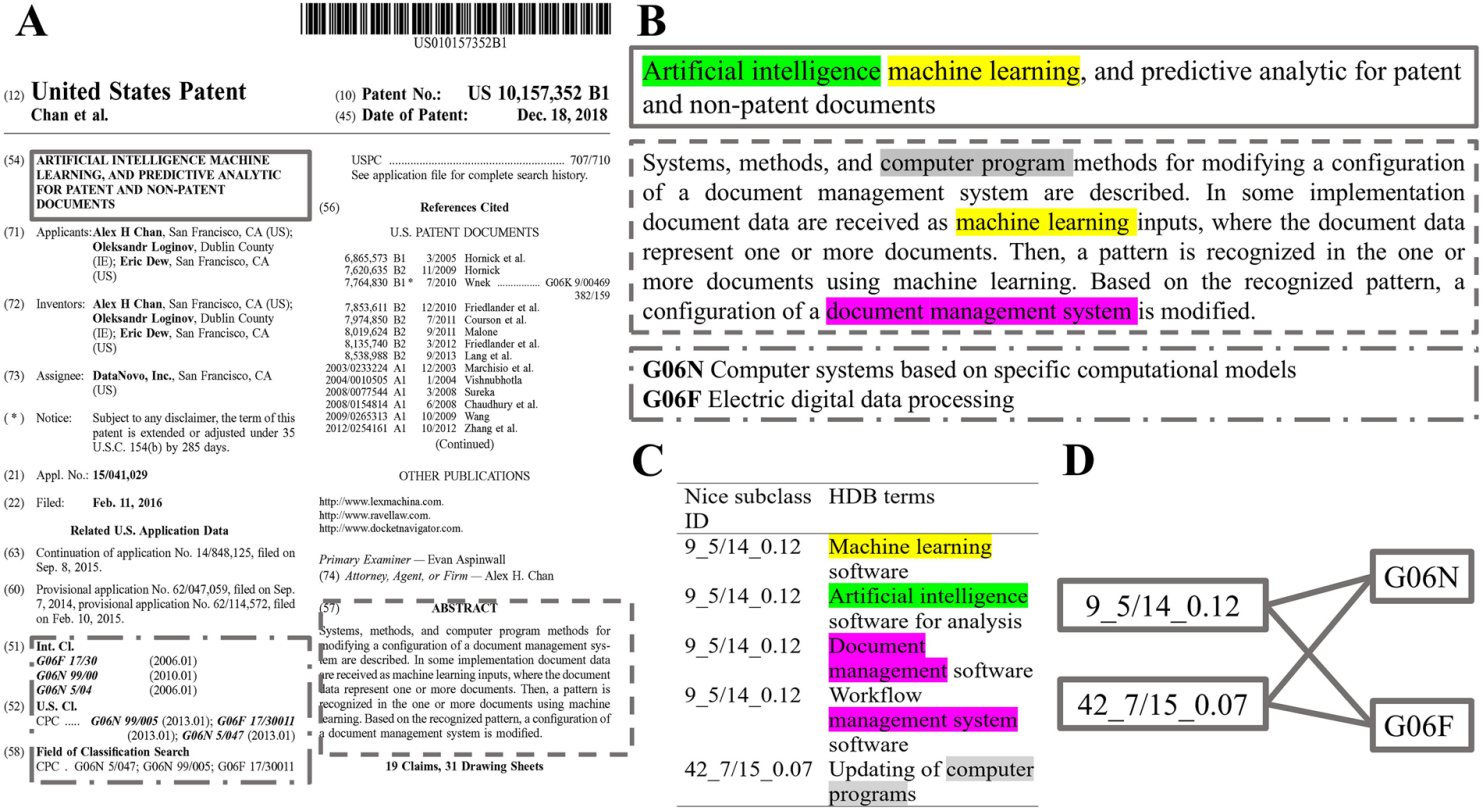
Bigram search strategy. Notes: Panel A illustrates a patent document from which we extract the text information. The solid and dashed boxes enclose the title and abstract, while the dot-dashed box highlights the CPC classes associated to the patent. Panel B zooms in on the three boxes. Panel C presents a sample of Nice subclasses along with several corresponding bigrams (highlighted), which were searched in the titles and abstracts of patent documents. The highlights in Panel B demonstrate the result of the bigram search. Panel D visualizes the results of the search strategy, linking two CPC codes with two Nice subclasses.

Figure 2 exemplifies how we matched bigrams in patents and trademarks. Panels A and B show a patent document from which we extract relevant information for our study, namely the title (solid box) and abstract (dashed line box). Note that searching in these fields, instead of searching in the full text of patent documents, is a common strategy in earlier empirical research^34–36^. In addition, the dot-dashed line highlights the CPC classes associated with the patent. Panel C includes a list of Nice subclasses and their bigrams, as searched in the patent document’s title and abstract. The highlights in Panel B indicate the bigrams matched in the patent document. Finally, Panel D visualizes the result of the search strategy, linking two CPC classes with two Nice subclass codes. The overall result of Step 3 was a weighted graph with 400,816 relations (656 CPC classes by 611 Nice subclasses), each weighted by the frequency of matches in the patent search.

In Step 4, we then translated the weighted graph of the bigram matches into probabilistic links between the CPC classes and the Nice subclasses. We first created a null expectation for the co-occurrence of CPC and Nice subclass, to consider relations only observed by chance. We followed the method suggested by Teece *et al*.^37^ and applied by others in empirical studies^9,38,39^. Formally, the z-score is defined as follows:

1 $${Z}_{i,j}=\frac{{O}_{i,j}-{E}_{i,j}}{{\sigma }_{i,j}}$$

where *O*_*i*,*j*_ denotes the frequency of bigrams of a Nice subclass *j* found in a patent title and abstract including a CPC technology code *i*, *E*_*i*,*j*_ denotes the expected co-occurrence as a null expectation capturing how often Nice subclass *j* would randomly occur in a patent title and abstract including a CPC technology class *i* and the standard deviation ensures standardized scores. The expected co-occurrence (*E*_*i*,*j*_) is given by:

2 $${E}_{i,j}=\frac{{n}_{i}{n}_{j}}{N}$$

where *n*_*j*_ is the total number of patents that include at least one bigram of the Nice subclass *j* in their title or abstract, *n*_*i*_ is the number of patents associated with the CPC technology code *i*, and *N* is the total number of patents. The standard deviation is calculated as:

3 $${\sigma }_{i,j}=\sqrt{{E}_{i,j}\left(1-\frac{{n}_{i}}{N}\right)\left(\frac{N-{n}_{j}}{N-1}\right)}$$

Intuitively, a positive z-score value indicates that the number of observed co-occurrences is higher than expected by chance. Hence, we established a concordance link between a CPC class and a Nice subclass whenever the z-score value was above zero.

**Fig. 3:**
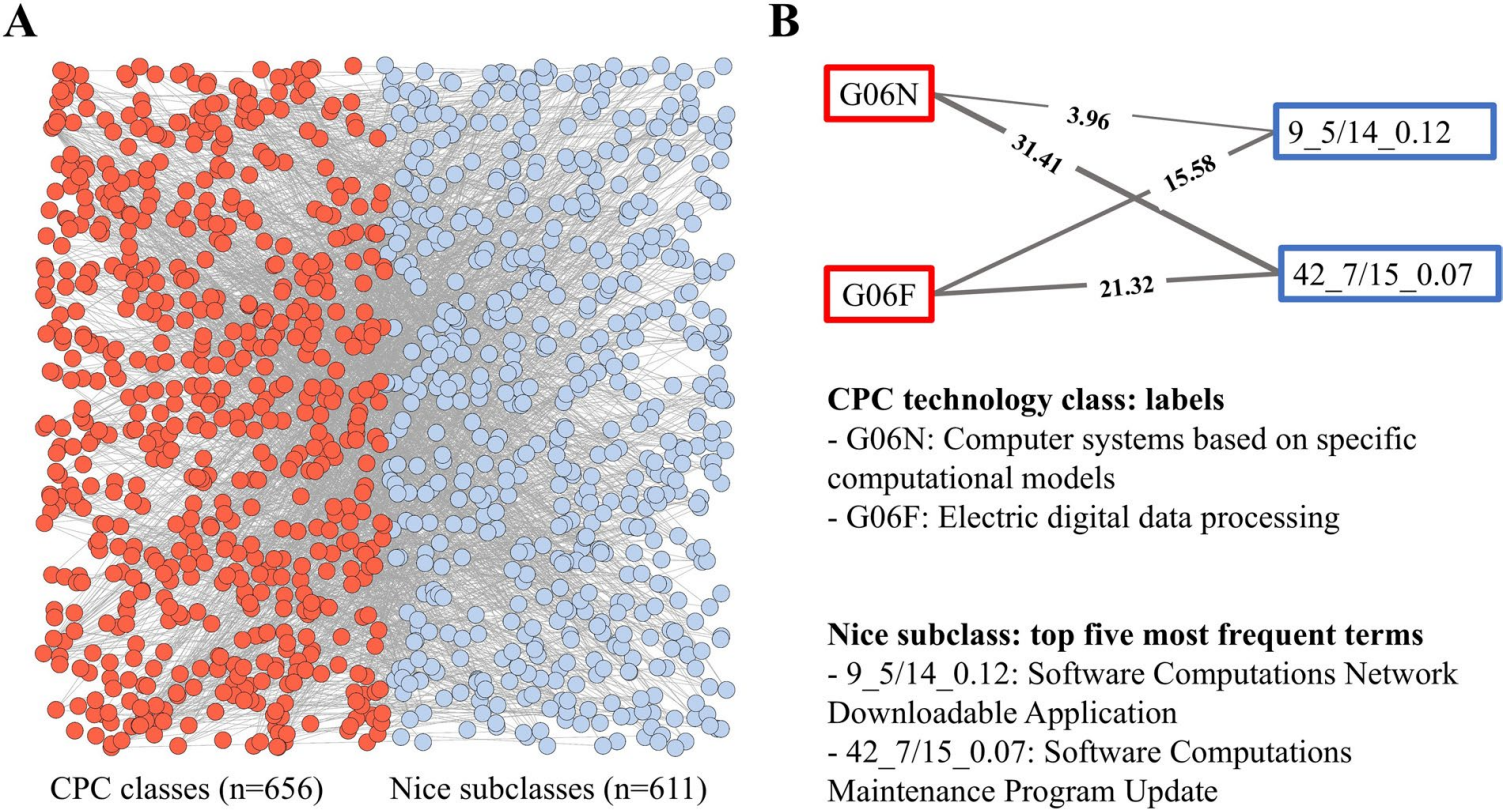
Illustration of the PAT2TM concordance. Notes: Panel A shows the concordance between CPC technology classes and Nice subclasses, identifying relationships through z-score values. For the sake of clarity, network links are displayed for relations with z-score values greater than 12.35 (the 95th percentile of positive z-scores). Panel B provides a closer look at two CPC classes and two Nice subclasses. It illustrates that each link in the concordance has a specific weight, allowing users to select links according to their own threshold.

In the final step, we constructed a concordance based on CPC-class and Nice-subclass pairs exhibiting statistically significant co-occurrence. This was done by selecting only those pairs with z-scores greater than zero. One can choose a higher value if interested in identifying stronger associations by specifying a threshold between 0 and the maximum observed z-score. After determining this threshold, we visualized the resulting PAT2TM concordance as a network, where CPC classes and Nice subclasses are represented as nodes and their associations as weighted edges (see Fig. 3).

The estimated z-score values range between -34.59 and 234.67 (median: -0.55 and mean: -0.66). Our results show that 10% of the CPC class-Nice subclass pairs had a z-score value greater than zero. These pairs were translated into links in our probabilistic concordance between patent and trademark classes. A substantial share of CPC classes (656 out of 662) and Nice subclasses (611 out of 616) have at least one link in the concordance.

Panel A of Fig. 3 visualizes the linkages between CPC technology classes and Nice subclasses as captured in our concordance. The visualization only shows the strongest links (with z-scores above the 95th percentile). When considering all links with positive z-scores, each technology class is connected to 62.85 Nice subclasses on average (median: 62) while Nice subclasses are linked to 67.48 technologies (median: 69). Panel B zooms in on two CPC classes and two Nice subclasses. The classes are connected by links whose weights correspond to the z-scores. For example, the technology class G06N (*Computer systems based on specific computational models*) has 82 relations with Nice subclasses, with varying intensities ranging from 0.01 to 31.41. The Nice subclass with the highest z-score includes the keywords *Software, Computations, Maintenance Program* and *Update*. In contrast, some technologies have significantly fewer relations to Nice subclasses. The CPC technology code C06F (*Matches; manufacture of matches*) has only one relation to a Nice subclass, probably due to the fact that this technology is obsolete.

The PAT2TM concordance includes all links between CPC classes and Nice subclasses with z-score values greater than zero. A key feature of our concordance is that the z-score values are also reported. As such, users can adapt their concordance to their needs and work with their own threshold. For example, setting a threshold of 2.5, instead of zero, means including 12,883 links (3% of all possible links instead of 10%).

Moreover, others can replicate our methodological approach (see Code availability) to estimate the concordance links for different time periods. This would allow investigating whether and how linkages between patented technologies and market applications have evolved over time. Importantly, the datasets used are publicly available. Hence, our method can be replicated as an unsupervised method that does not require access to specific technology-level or firm-level databases. We produced the concordance using EPO EUIPO data but our approach can be applied to data from other national offices, such as the USPTO and other offices that align with the Madrid protocol and rely on English as an official language.

#### Sensitivity Analysis on the Concordance

Our validation strategy (and its validation) is centered on the final outcome. The main reason for doing so has to do with the lack of obvious external benchmarks to validate the intermediate steps that we have described before. The aim of this subsection is to provide a set of tests to evaluate the sensitivity of our concordance to changes in the intermediate steps of our methodology. In particular, we consider how the concordance behaves under alternative specifications in steps 2, 3, and 4 (see Fig. 1). In addition, we evaluate the temporal and spatial stability of our concordance.

To validate the robustness of the clustering procedure in Step 2, we assessed the sensitivity of the results to the choice of the number of clusters *K*. Although the elbow method provides a data-driven criterion for selecting *K*, clustering outcomes may still depend on this choice. We therefore test whether HDB terms are consistently grouped together across varying numbers of clusters. Specifically, for each Nice class, we repeat K-means clustering with *K* + 1 and *K* − 1 clusters and compare these alternative partitions against the baseline clustering. To quantify the similarity between clusterings, we use the Adjusted Rand Index (ARI), which measures the agreement between two partitions while correcting for chance. The ARI ranges from  − 1 to 1, where 1 indicates perfect agreement, 0 corresponds to random assignment, and negative values indicate less agreement than expected by chance. We obtain ARI values of 0.719 (for *K* − 1) and 0.723 (for *K* + 1), which indicates that the clusterings are not in disagreement and that the identified Nice subclasses are not highly sensitive to the choice of *K*. The semantic grouping of HDB terms remains relatively stable.

Regarding Step 3, we test alternative n-gram specifications by comparing unigram, bigram, and trigram-based patent text searches. This step highlights an inherent trade-off between contextual richness and the ability to generate sufficient matches. We construct alternative concordances using unigram and trigram searches and compare them to the baseline bigram specification. The unigram approach produces a dense many-to-many network of links, but these links largely ignore context, failing to capture meaningful relationships between technologies and trademarks. For example, a stem such as “chemic” can match a wide range of patent texts, whereas a bigram such as “chemic reactor” provides clearer, more relevant context. In contrast, the trigram-based concordance results in a very sparse network with only a few links between classes. While trigrams offer highly specific context, they significantly reduce the number of matches. For instance, a trigram such as “laboratori chemic reactor” appears only rarely in patent texts, whereas “chemic reactor” captures similar technological content with a much larger set of observations. A combined specification that uses both bigrams and trigrams in the patent text search produces results that are close to the baseline search (bigram only), in fact the agreement between both approaches has an F1 score of 0.86.

To test Step 4, we used an alternative method to ensure that our concordance is not sensitive to the z-score specification. We followed the approach of Neffke *et al*.^40^, who approximated skill-relatedness structures using labor flows between industries. In our context, we treated the co-occurrence of CPC codes and Nice subclasses as a bipartite network, where CPC codes and Nice subclasses constitute the two node sets, and weighted edges capture the frequency with which CPC codes and Nice subclasses co-occur, based on bigram matching in patent texts. We define the relatedness measure as:

4 $${R}_{ij}=\frac{{F}_{ij}\,F}{{F}_{i}\,{F}_{j}}$$

where *F*_*ij*_ denotes the co-occurrence frequency of CPC code *i* and Nice subclass *j*, *F*_*i*_ = ∑_*j*_*F*_*ij*_ represents the weighted degree centrality of CPC code *i*, *F*_*j*_ = ∑_*i*_*F*_*ij*_ denotes the weighted degree centrality of Nice subclass *j*, and *F* = ∑_*i*_∑_*j*_*F*_*ij*_ is the total sum of all co-occurrences. Since the distribution of *R*_*ij*_ is right skewed on the interval [0, *∞*), we apply the following transformation:

5 $${\bar{R}}_{ij}=\frac{{R}_{ij}-1}{{R}_{ij}+1}$$

The correlation between *Z*_*ij*_ values, computed using the equation described in Step 4, and ${\bar{R}}_{ij}$ is high, with a Spearman rank correlation coefficient of 0.86, indicating a strong positive association between the two measures.

**Fig. 4:**
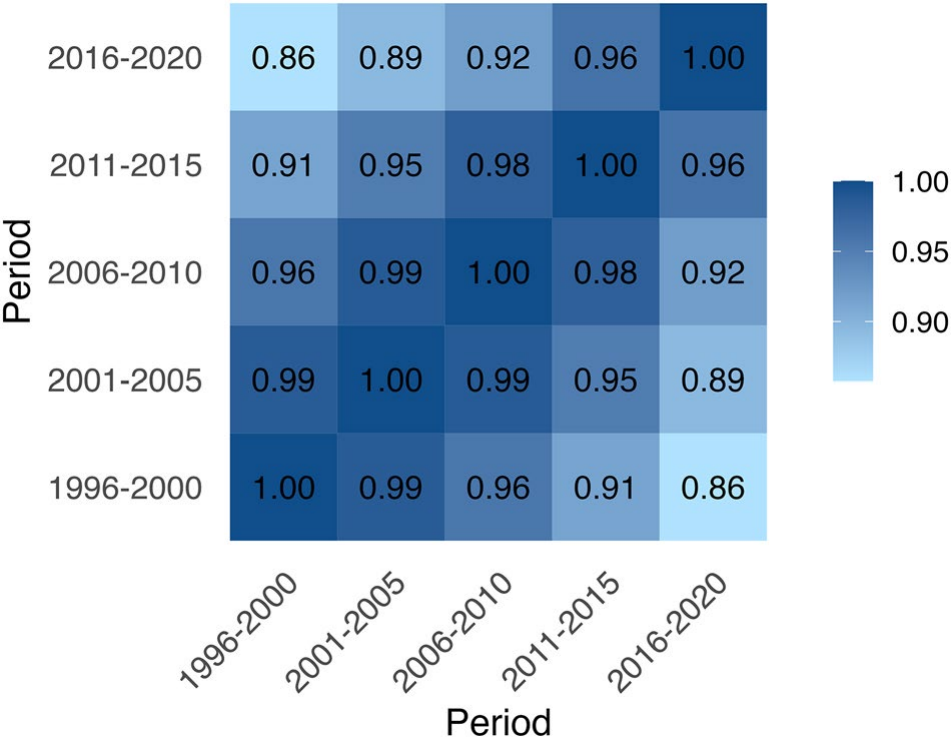
Stability of the PAT2TM concordance over time. Notes: The heatmap reports Pearson correlation coefficients between z-score values computed over five consecutive time windows (1996-2000 to 2016-2020). Correlations are highest between adjacent periods (typically 0.96-0.99), indicating strong temporal persistence in the concordance structure.

Because the concordance is derived from aggregated data and was created using the PATSTAT 2020 edition, we assess its temporal stability. In addition, such validation test provide useful information to evaluate whether reporting lags could be an important issue as well. Figure 4 shows the intertemporal correlation of the concordance measure. As expected, similarity is strongest between adjacent time windows, with Pearson’s correlation coefficients generally exceeding 0.95. This indicates a high degree of short-run stability in the relative alignment between patent and trademark classes. As we compare periods that are further apart, these correlations gradually weaken. In particular, the association between the earliest (1996-2000) and the latest (2016-2020) periods is approximately 0.86. This suggests that the concordance is quite stable, yet it evolves meaningfully over time. This pattern is consistent with what one may expect giving the slow-moving nature of technological change, market composition, and classification practices.

Finally, we evaluate how our concordance varies across alternative definitions of patent families. We used the DOCDB family identifiers and therefore only included singleton patents (i.e., families with a single patent application). This approach may introduce potential biases, as singletons are more prevalent in certain countries, particularly in China, Japan, and South Korea, given differences in patenting regimes and filing strategies. We conducted a robustness check using geocoded patent data developed by de Rassenfosse *et al*.^41^, covering worldwide first filings between 1980 and 2014. We estimated *Z*_*ij*_ (see Equation 1) using the full dataset and compared it to an alternative specification, ${Z}_{ij}^{\,{\rm{limited}}}$, where we excluded all patents with at least one inventor located in China, Japan, or South Korea. The resulting measures are highly correlated (Pearson correlation coefficient: 0.98), indicating that our approach is not affected by the spatial distribution of singleton patents.

## Data Records

Our concordance is available as PAT2TM Database^42^, archived at the Harvard Dataverse site, hosted by Harvard University. The “PAT2TM.csv” file, within the “PAT2TM Database” folder, contains six columns and 41,228 rows. Each row represents a link between a CPC technology class and a Nice subclass. Columns provide the following information:

- **CPC**_**4digit** provides the Cooperative Patent Classification (CPC) classification at the 4-digit level as described in the method section (https://www.epo.org/searching-for-patents/helpful-resources/first-time-here/classification/cpc.html).
- **Nice**_**subclass** provides the Nice subclass developed based on the HDB goods and services terms described in the methods section.
- **Zij** represents the positive z-score values in Equation 1. It ranges between 0.000015 and 234.67 (mean: 3.26, median: 1.36, standard deviation: 6.99).
- **CPC**_**4digit**_**label** represents the label of CPC technology codes at the 4-digit level provided by CPC classification (https://www.epo.org/searching-for-patents/helpful-resources/first-time-here/classification/cpc.html).
- **Nice**_**subclass**_**label** provides descriptive labels that are comparable to CPC classification labels. To create these labels, we used a generative AI model (GPT-4) to suggest summaries based on the goods and services terms associated with each Nice subclass. These suggestions were then manually inspected for all subclasses. The labels correspond closely to the characteristic terms of each subclass and the most frequent stems, as further illustrated in the description of the Nice_subclass_keyword variable.
- **Nice**_**subclass_keyword** consists of the most frequent stems in each Nice subclass. For clarity, we padded each stem with the most frequent suffix for the corresponding stem.

Moreover, we provide another dataset to link the Nice subclasses to the HDB goods and services terms (see Properties of the Trademark data) which we will refer to as the HDB2Nicesub dataset. The ‘HDB2Nicesub.csv’ file contains six columns and 58,155 rows. Each row represents a unique HDB goods and services term linked to a Nice subclass. Nice subclasses cover, on average, 94.44 (median:68) HDB goods and services terms. This dataset can be linked to the PAT2TM Database via *Nice_subclass*. Columns of HDB2Nicesub provide the following information:

- **Nice**_**subclass** (see above, same variable as in the first dataset).
- **Nice** reports the Nice class, as described in the method section (https://euipo.europa.eu/ec2/).
- **Type** shows whether the Nice_subclass is related to a good or a service (https://euipo.europa.eu/ec2/).
- **Nice**_**subclass**_**label** (see above, same variable as in the first dataset).
- **Nice**_**subclass**_**keyword** (see above, same variable as in the first dataset).
- **HDB**_**term** consists of the HDB (the Harmonised Database) goods and services terms that have been accepted by all national intellectual property offices in the European Union (EU) and used by the EUIPO (European Union Intellectual Property Office) (https://euipo.europa.eu/ohimportal/en/harmonised-database).

## Technical Validation

We validated our concordance using two validation datasets, both of which provide alternative approaches to link between technologies/patents and products/trademarks. Specifically, we tested the reliability of GPT-4 as an informed, uneducated assistant using two ‘real-world’ datasets with information on connections between technologies and markets. It is important to note that both validation sets are much smaller than the patent and trademark datasets we used to develop the concordance.

The first validation dataset is the EUIPO patent-trademark bundles^43^. Firms often file patents and trademarks to protect different features of the same product. Such patent-trademark bundles represent a good benchmark for flagging actual patent-trademark linkages within the same innovation project. The data from the EUIPO patent-trademark bundles includes firms’ patent and trademark filings between 2014 and 2015^43^. This manually curated sample contains 76,202 EPO patents and 98,257 EUIPO trademarks filed by 63,286 firms. To derive links as precisely as possible, we took a conservative approach and selected patent and trademark documents whose owners filed for only one patent and one trademark within six months. This approach avoids bias from linking multiple unrelated patents and trademarks, which may increase the number of false negatives. We identified 6,827 trademark-patent pairs to be used as a comparison benchmark. Next, we paired the label of the CPC technology classes mentioned on a linked patent document and the goods and services descriptions included in the trademark document (13,503 pairs).

**Fig. 5:**
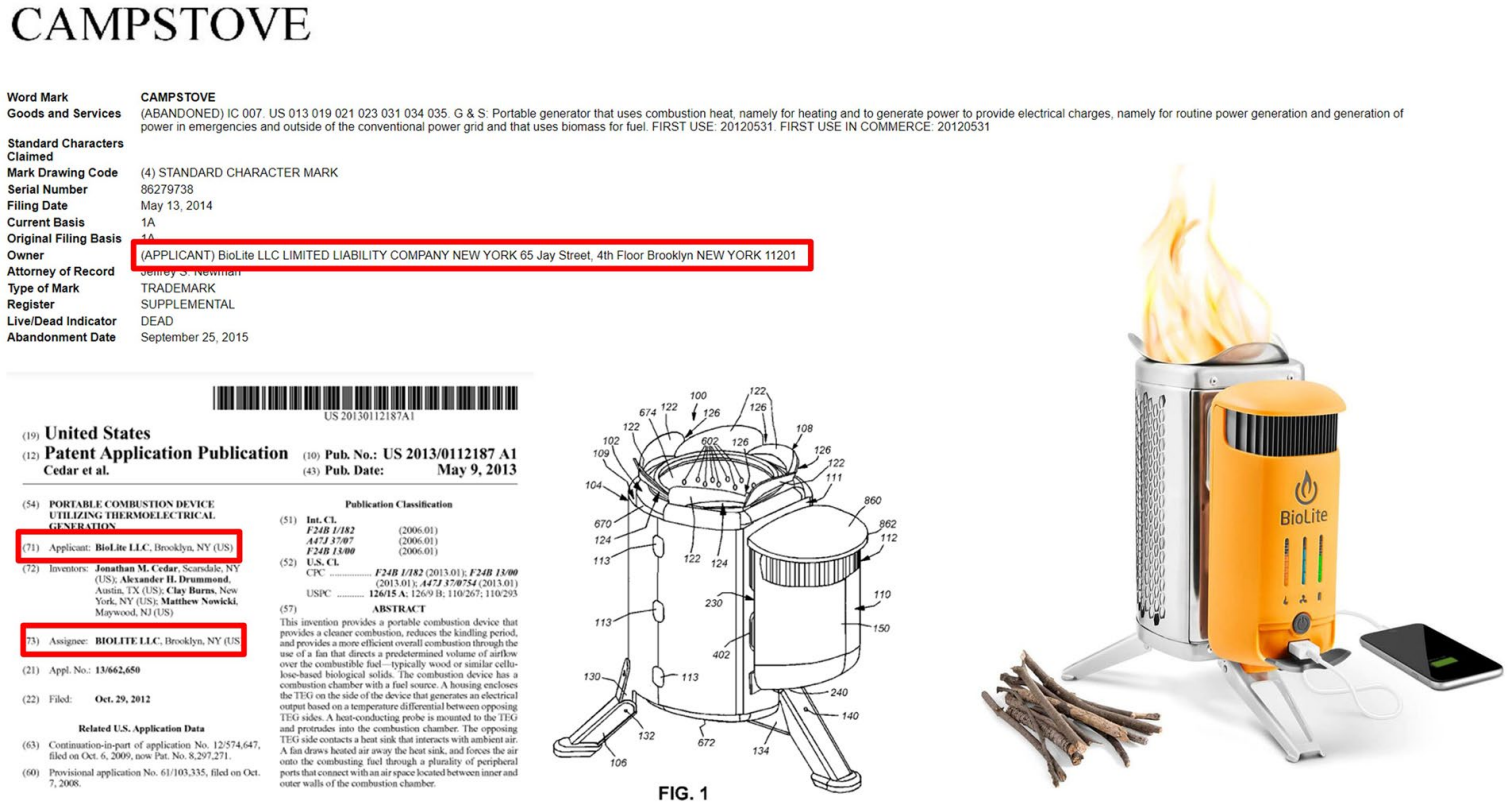
An example of IProduct-USPTO patent-product-trademark triad. Notes: This figure shows that a product (e.g., BioLite) is linked to a USPTO patent in the IProduct dataset. We found a trademark for the same product through string matching in the USPTO patent dataset. Next, we paired the CPC technology class labels and goods and services descriptions mentioned in the linked patents and trademark documents.

The second validation dataset is IProduct, which results from a project linking patents to products. This database contains information on 353,649 patent-product relationships (https://iproduct.io/app/public/page/home). We used this dataset to construct patent-product-trademark triads to derive trademark-patent pairs. We created the triads by matching product names to trademarks as well as to patent and trademark applicant names. This process involved two steps. First, we applied a combination of algorithmic and manual string matching techniques to align trademark applicants with patent applicants. Second, we matched product names appearing in both the IProduct database and the USPTO trademark data. It is important to note that we intentionally avoided using the EUIPO data employed to construct the concordance, ensuring that the validation exercise remains fully independent of the concordance’s underlying data.Through this approach, we identified 1,973 patent-product-trademark triads in total (see Fig. 5).

For the validation exercises, we built on recent advances in large language models (LLMs). LLMs leverage neural networks with a transformer architecture^44^. They are trained on extensive text datasets to classify, analyze, and generate human-like text and handle complex language tasks by predicting and assembling text sequences^45,46^. In economics and social sciences, researchers have used LLMs in novel theory generation^47^, synthetic data production^48,49^, social dynamics modeling^50^, and deductive coding of unstructured data^51,52^. These models are also increasingly finding application in innovation research^53^.

Earlier studies suggest that using a pre-trained generative AI model such as OpenAI’s GPT for ‘straightforward’ cognitive tasks can result in satisfactory performance and significant accuracy^46,49,54^. Recent evidence indicates that GPT-4o outperforms earlier versions, such as GPT-3.5, in predicting human interests and performing cognitive tasks^55^ and that GPT-4 performs similarly to GPT-4o^46,49^. Accordingly, we validated the patent-to-trademark concordance with OpenAI’s GPT-4 (the gpt-4-turbo model). GPT-4 was not trained on our concordance, hence we could use it to validate it through out-of-sample predictions. To evaluate both the quality of our concordance and the reliability of GPT-4 as an informed assistant, we first sampled 1,000 pairs of CPC technology labels and goods and services descriptions from each validation dataset. We also generated a benchmark of descriptions of randomly selected goods and services. Table 2 shows three examples from each dataset. Next, we evaluated whether GPT-4 agrees with the relationships between technologies/patents and products/trademarks in these datasets using the following prompt:

I give you a description of a technology: descriptor: *CPC technology class labels*. Next, I give you the descriptions of two distinct market product keywords: to 1: *Goods and services description* and to 2: *Randomly selected goods and services description*. If description is more related to to1, give a response Result: 1; otherwise, give a response Result: 0. Provide output as Result: 1 or Result: 0 without further token creation.

**Table 2:** Lists of CPC class labels, goods and services descriptions, and randomly selected goods and services descriptions used for the two validation sets.

CPC Technology Class labels	Goods and Services descriptions	Randomly Selected Goods and Services descriptions
Valves; Taps; Cocks; Actuating-Floats; Devices for Venting or Aerating	Valves	Computers; Computer hardware
Microorganisms or Enzymes; Compositions thereof; Propagating, Preserving or Maintaining Microorganisms; Mutation or Genetic Engineering; Culture Media	Chemicals	Footwear
Motor Vehicles; Trailers	Motors	Blouses
Optical Elements, Systems, or Apparatus	Accent lights for indoor use; LED lighting fixtures	Electric toothbrushes
Electric Digital Data Processing	Downloadable computer software and manuals sold as a unit, to create installation programs and intelligent file handling applications	Trailer tail
Preparations for Medical, Dental, or Toilet Purposes	Cosmetic bags sold empty	Lasers, optical amplifiers, devices for providing a source of amplified spontaneous emission

**Fig. 6:**
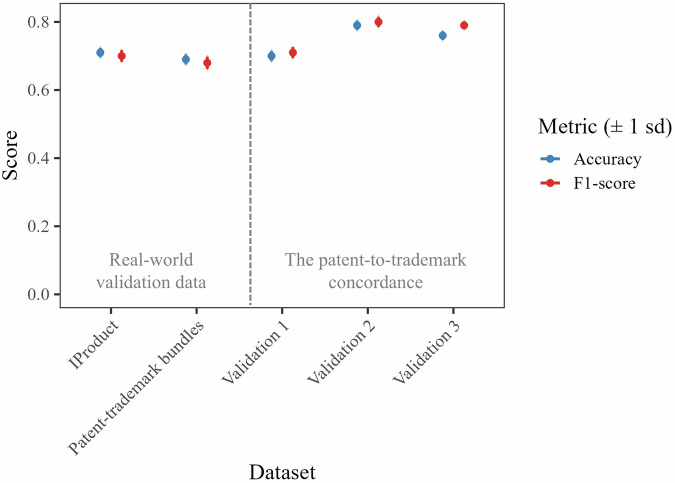
Agreement of the two validation datasets and the PAT2TM concordance validations with GPT-4.

We used the accuracy and F1-score to measure the performance. Accuracy measures the proportion of correct predictions (both true positives and true negatives) relative to the total number of predictions. The F1-score is the harmonic mean of precision and recall. The result of this exercise suggests an acceptable agreement for each validation dataset. Namely, the validation dataset 1 (EUIPO’s patent-trademark bundles) achieved an accuracy of 0.69 and an F1-score of 0.68. The validation dataset 2 (IProduct) resulted in an accuracy of 0.71 and an F1-score of 0.70. We did not observe a significant change in performance metrics when incorporating examples into the prompts, switching from a zero-shot to a few-shot approach Fig. 6.

We then applied the same prompt to three samples of links from our PAT2TM concordance. In Validation 1, we sampled 1,000 random pairs of CPC classes and two sets of Nice subclasses with *Z*_*ij*_ values greater and less than zero (500 pairs each). The expectation was that GPT-4 would identify pairs of technology and market classes with positive *Z*_*ij*_ values as more related than those with negative *Z*_*ij*_ values. In validation 1, we achieved an accuracy of 0.70 and an F1-score of 0.71.

In Validation 2, we sampled 1,000 pairs of CPC classes and Nice subclasses where *Z*_*ij*_ values were greater than the 75^th^ percentile and included additional Nice subclasses linked to the same technologies but with negative *Z*_*ij*_ values. As expected, the performance metrics improved substantially, achieving an accuracy of 0.79 and an F1-score of 0.80.

The first two validations focused on validating the sign of the *Z*_*ij*_ values. Additionally, we performed Validation 3 to explore the strength of positive z-scores. We first selected all pairs with *Z*_*ij*_ values greater than zero. Next, we randomly selected 1,000 CPC class-Nice subclass pairs with *Z*_*ij*_ values greater than the 90^th^ percentile (strong positive relationships) and less than the 10^th^ percentile (weak positive relationships). The results indicated that GPT-4 and the concordance aligned in identifying the links, with an accuracy of 0.76 and an F1-score of 0.79.

Overall, the results from all three validations align closely with our earlier tests conducted on the real-world validation datasets. We set the temperature argument in GPT-4 to zero across all validation tests for consistent and deterministic results (i.e., removing randomness by making the model always choose the most probable next token). By sharing our validation Python script (see Code availability), we enable other researchers to replicate our validation exercise easily.

## Usage Notes

In the PAT2TM Data folder, we provide open access to the patent-to-trademark concordance (‘PAT2TM.csv’) and the complementary dataset (’HDB2Nicesub.csv’) for linking Nice subclasses to a wide range of goods and services terms. Downloaded datasets can be easily loaded, analyzed, and merged with any statistical software tools. To visualize the concordance as a network (see Fig. 3), we recommend using R packages like igraph or network packages, Python igraph or NetworkX libraries, or Gephi (an open-source software platform).

The PATSTAT dataset is not publicly accessible and requires purchase for use (https://shop.epo.org/en/Data-and-services/Bulk-data-sets/PATSTAT-Global/p/PS).

A complete list of HDB terms is publicly available at the EUIPO’s website (https://euipo.europa.eu/ec2/).

Regarding the two validation datasets, the EUIPO patent-trademark bundles^43^ and IProduct (https://iproduct.io/app/public/page/home) are not publicly available. Those interested in accessing them should contact the researchers who developed the datasets directly. The EUIPO trademark data is available upon requesting a license.

## Data Availability

Our concordance is available as PAT2TM Database^42^, archived at the Harvard Dataverse site, hosted by Harvard University. This database can be accessed at: 10.7910/DVN/JD7JIL

## Code availability

We share the script ‘PAT2TM.ipynb’, which allows researchers to replicate our concordance and apply our method to create concordances using other datasets with similar properties. The script provides codes for each step of developing the concordance described in the method section and Fig. 1. We also provide the script (‘Validation.ipynb’ and the needed file). The scripts can be used in a Jupyter Notebook or Google Colab.
